# Wheelchair users, access and exclusion in South African higher education

**DOI:** 10.4102/ajod.v6i0.353

**Published:** 2017-09-08

**Authors:** Desire Chiwandire, Louise Vincent

**Affiliations:** 1 Department of Political and International Studies, Rhodes University, South Africa

## Abstract

**Background:**

South Africa’s Constitution guarantees everyone, including persons with disabilities, the right to education. A variety of laws are in place obliging higher education institutions to provide appropriate physical access to education sites for all. In practice, however, many buildings remain inaccessible to people with physical disabilities.

**Objectives:**

To describe what measures South African universities are taking to make their built environments more accessible to students with diverse types of disabilities, and to assess the adequacy of such measures.

**Method:**

We conducted semi-structured in-depth face-to-face interviews with disability unit staff members (DUSMs) based at 10 different public universities in South Africa.

**Results:**

Challenges with promoting higher education accessibility for wheelchair users include the preservation and heritage justification for failing to modify older buildings, ad hoc approaches to creating accessible environments and failure to address access to toilets, libraries and transport facilities for wheelchair users.

**Conclusion:**

South African universities are still not places where all students are equally able to integrate socially. DUSMs know what ought to be done to make campuses more accessible and welcoming to students with disabilities and should be empowered to play a leading role in sensitising non-disabled members of universities, to create greater awareness of, and appreciation for, the multiple ways in which wheelchair user students continue to be excluded from full participation in university life. South African universities need to adopt a systemic approach to inclusion, which fosters an understanding of inclusion as a fundamental right rather than as a luxury.

## Introduction

Accessibility must be at the front and centre of student politics. When we say we need to make our universities more accessible to students we’re including race, social status, disability and a host of things that comprise our identity. Last year we saw the Rhodes Must Fall, Fees Must Fall and Patriarchy Must Fall movements, challenging things like racism, classism and sexism. But I don’t think we’ve seen a radical or a strong conversation about ableism or about disability. … That means we have not addressed the issue in totality. (University of Cape Town student activist Busi Mkhumbuzi, cited in Hendricks 2016)

Poorly designed physical environments exclude persons with disabilities (PWDs) from participating in mainstream society (DSD, DWCPD & UNICEF 2012:20). Wolanin and Steele (2004), for example, point to ‘curbs and stairs that cannot be navigated by wheelchairs or mounted by the physically frail; [the unavailability of] tactile maps for the blind, and no TTY^1^ phones for the deaf’ (p. 53). Lack of elevators, ramps, automatic doors, Braille signage and telecommunication devices are among the more obvious factors that deter and restrict the equal participation in various spheres of public life of PWDs (Gal et al. 2010:91). As Howell and Lazarus (2003:68) have argued, it is a central requirement of respect for diversity that ‘physical barriers that limit mobility and thus access to institutional services for some disabled students, especially physically disabled and blind students’ be eliminated.

In apartheid South Africa, the education of students with disabilities (SWDs) was low on the priority list of the National Party government. Little attention was paid to developing the built environments of educational institutions in such a way as to include SWDs – particularly students with physical disabilities (SWPDs). Post-1994 saw the advent of democracy and the enactment of a new Constitution (Section 29[1][a]) that guaranteed everyone, including PWDs, the right to education, and inclusive education policies such as the *Education White Paper 6, Special Needs Education: Building an Inclusive Education and Training System* impose an obligation upon all higher education institutions (HEIs) to ensure that there is appropriate physical access for all learners. South Africa’s 2008 *National Building Regulations and Building Standards Act* provides for minimum standards of accessibility to be applied in the design of new buildings. South Africa is also a signatory to the United Nations Convention on the Rights of Persons with Disabilities (UNCRPD) 2006 (see DSD et al. 2012:19), which entails ‘an obligation to take proactive measures to ensure that the rights of persons with disabilities are promoted and protected’ both in higher education (HE) and work environments (South African Human Rights Commission 2012:1). Among other things, this should take the form of ‘ensur[ing] an inclusive education system at all levels’ including HEIs, which should be in line with the provisions of Article 24 of the UNCRPD.^2^ Article 24 also obliges states to provide reasonable accommodations and appropriate support services tailored to individuals’ educational needs as a measure of ensuring that PWDs can participate effectively in a free society.

In 2007, the South African government ratified the UNCRPD. Article 9 of the UNCRPD obliges HEIs to be physically accessible to PWDs by applying the accessibility principles of ‘universal design’ and ‘inclusive design’. Accessibility refers to ‘the degree to which an environment, service, or product allows access by as many people as possible, in particular people with disabilities’ (World Health Organization [WHO]; World Bank 2011:303). ‘Accessibility’ entails making it possible for ‘persons with disabilities to live independently and participate fully in all aspects of life’. Signatories to the Convention are thus obliged to

take appropriate measures to ensure to persons with disabilities access, on an equal basis with others, to the physical environment, to transportation, to information and communications, including information and communications technologies and systems, and to other facilities and services open to or provided to the public.

Obstacles and barriers to accessibility – for example, in relation to buildings, roads, transportation, housing and outdoor facilities – must be identified and eliminated. In many countries, many social spaces are inaccessible to wheelchair users; however, the passage of Article 9 on Accessibility of the 2006 UNCRPD has imposed obligations on signatory countries with respect to accessibility for wheelchair users including the accessibility of social spaces such as cafeterias and restaurants.

The post-apartheid government encouraged universities to conduct access audits ‘wherein the existing facilities are assessed and suggestions provided for further improvement’ (Agarwal 2012:56). In its Green Paper of 2012, the Department of Higher Education and Training (DHET) made a commitment to determine the financial needs of various disability units and to allocate resources based on the needs of each unit (DHET 2013, cited in Lourens 2015:116). The following year, in the *White Paper* of 2013 it became clear that this commitment was not empty with the provision of funding for infrastructure audits at each of the country’s 23 public universities and an allocated R130 million for improving accessibility on campuses (DHET 2013, cited in Lourens 2015:116).

In practice, however, South Africa’s progressive legislation has had minimal impact on improving campus access for SWDs with many buildings remaining inaccessible and in principle commitments to accessibility and inclusivity remain unrealised. Howell and Lazarus (2003), for instance, have pointed out that

whilst new buildings and facilities must now meet the requirements of the National Building Regulations of 1986, these regulations are not sufficiently enforced and many new buildings built on campuses since 1986 remain inaccessible, particularly to wheelchair users. (p. 69; see also FOTIM 2011:11)

There have been numerous instances of admission to public universities in South Africa being denied to SWDs on the grounds that they do not have appropriate facilities to accommodate these students. For example, in November 2013 Nelson Mandela Metropolitan University denied admission to three visually impaired students. In January 2015, a wheelchair user’s application was turned down by Tshwane University of Technology on the grounds that the university was not physically accessible to wheelchair users. In Losinsky et al.’s (2003) study that investigated the physical accessibility to wheelchair users of one South African university, participants expressed concerns over the inaccessibility of some campus buildings, including facilities such as toilets (see also Matshedisho 2010:732).

Most South African universities have disability units that are charged with the responsibility of protecting and promoting the interests and rights of SWDs. For the purpose of this study, we interviewed 28 disability unit staff members (DUSMs) at 10 different South African universities to gain an insight into why so little progress has been made with regard to reconfiguring South African university campus environments in order to ensure equal and full access for SWDs. The participants described a range of challenges that they face with calling universities to account for failing to promote full access and participation in HE for SWDs.

Initial coding of these interviews revealed commonalities in the way in which DUSMs based at 7 of the 10 universities in the larger study spoke at length about challenges faced by wheelchair user students (WUSs) in particular on their campuses because of the physical inaccessibility of the built environment and a lack of wheelchair-adjusted transport. The data were therefore disaggregated and for the purpose of the present paper, the interviews with the 13 DUSMs who focused on issues such as ramps, railings, stairs, staircases, curbs, sidewalks, narrow passages, pathways and parking spaces – all of which are particularly relevant to wheelchair users – formed the focus.

### Ethical consideration

Ethical clearance was obtained from all sampled institutions.

## Findings

South African universities have a long way to go with regard to putting into practice the on-paper commitments that the country has made to creating educational environments that are accessible and universally inclusive. The principle of universal design (UD) enjoins building planners, engineers, architects and the like to design ‘buildings that are suitable for all users’ (Imrie & Hall 2001:335) rather than taking the approach of adding to or adapting physical spaces designed for non-disabled people (Chard & Couch 1998:605). Environments must, as David Mitchell (2010) has argued, ‘be usable by the greatest possible range of people, to the greatest extent possible, without the need for subsequent adaptation or specialised design’ (p. 13). The principle of ‘inclusive design’ (Chard & Couch 1998:607) moreover points to the need to make physical environments accessible not only to students of one type of disability but rather to those with diverse disabilities. Inclusive design has to do with designing environments in such a way that diverse people will benefit and have their quality of life enhanced by living and working in such environments (see Chard & Couch 1998:607).

The DUSMs interviewed for this study identified a number of stumbling blocks preventing full and equal access to buildings, facilities and other campus spaces for wheelchair users in particular. These include appeals to the preservation and heritage as a justification for failing to modify older buildings, ad hoc and quick fix approaches to creating accessible environments with a lack of consultation with PWDs, the failure to address and prioritise issues such as access to toilets, libraries and transport facilities for wheelchair users, the failure on the part of universities to proactively create welcoming environments where all students feel they belong and are able to integrate socially and the relative powerlessness of DUs to call those in authority to account for failing to take steps to make progress towards overcoming these challenges.

### The preservation of historical heritage as a justification for exclusion

‘One of the critical challenges will be our facilities, this university actually was built 49 years ago so at that stage in terms of the Building Regulations there was never actually any urgency to make our facilities you know to be accessible. So, as a result, of that then, to comply, you need huge injection of resources so that you make your facilities accommodating you know your lifts, your access to offices, access to ablution facilities, your steps, your ramps, your parking bays, you know your stairs. So, there needs to be a huge amount of resources that needs to go into this now. It’s better here because some of our inaccessible building are not heritage sites. It becomes very difficult if you have that kind of buildings because then there is the Heritage Act that you need to preserve the building on the other hand and you know there are also those kinds of challenges.’ (Participant 1, male, 52 years)

The South African Heritage Resources Agency and National Heritage Council^3^ govern the conservation, protection and promotion of heritage resources (Parliamentary Monitoring Group 2015). Older buildings on some university campuses are considered as falling within the ambit of cultural heritage requiring protection and preservation. DUSMs pointed out that the idea of preservation was frequently used as an excuse to justify the failure on the part of universities to commit funds to making older buildings accessible to wheelchair users.

‘I would say protecting the historical heritage is partly one of the reasons why our buildings are inaccessible. I submitted a report saying this building and this building, there needs to be a lift or a ramp, so the first thing they would say is like it’s a ‘historical building’, but they don’t know that historical buildings can also be adjusted in some way, so that’s part of the problem.’ (Participant 2, female, 36 years)

‘Old buildings should be renovated in such a way to make them more accessible. … This campus is over 100 years old so we have old buildings … and 70s and they are not accessible at all and the problem being, so you see this building [*showing me the picture*] it was built in the 1930s so it’s a national heritage building so we cannot do anything at the front of the building, … now we must do something, make plans to do something at the back. So we are working with the new Building Regulations which guides you in how an accessible bathroom must look like. They have specifications on heights, lengths and breadths and everything. So we are looking at that. Slowly, but surely, it’s difficult because we [*are*] wanting things done as quickly as possible, but your guys on the other side of physical and infrastructural planning – there are other things that they are focusing on then disability gets forgotten.’ (Participant 3, male, 36 years)

‘If people keep on using excuses of historical buildings, and denying them access because of history, it will get to a point where disabled students demand the breakdown of historical building because you are not giving them access because that’s where we gonna go to at the end if they keep on using that excuse. So, negotiate and find a solution together with them otherwise you gonna get a Rhodes Must Fall type of situation where the disabled students will say we are fed up now you saying it’s because of this historical building, what is more important, the fact that historical building or the fact that as a wheelchair user I can’t get to my class.’ (Participant 4, female, 46 years)

### The cost of universal access as a justification for exclusion

As Participant 5’s comments make clear, the heritage argument is often tied closely to arguments about competing resources and resource constraints and without clear commitments to universal access on the part of university management DUSMs often find it difficult to ensure that disability policies are implemented in practice.

‘Making universities accessible can be done, but there has got to be a political will, there has got to be the will to spend money. Because you know at the university there is always competing claims there is never enough money for anything, everybody always think that they deserve that money whether it’s for their nuclear programme, or their medical transplant programme, or their African language programme so disability access … has to fight with all those other kind of competing claims on the university’s resources and of course nobody, it’s very difficult to persuade people that disability is that [*loud voice*] disability access is that important … and that is a real problem. Because if you are not disabled you don’t really get, until you have sat at the bottom of stairs in a wheelchair and thought how am I am I gonna get up these stairs … That’s only when it becomes something that people only get, but if you can run up the steps it’s easy to look around you and say where are these disabled people you are talking about, I don’t see anybody around. You know we often found that, they aren’t in a wheelchair where are they? Should we spend million making this place accessible for two people, you know and people say there are much more important things that we need to be doing. For instance, a movie theatre, it’s gonna cost me 10 million Rands to make this movie theatre accessible, but how many wheelchair users will have to buy tickets before I get my money back. Those are the kinds of arguments that ordinary people use. And you know disabled people aren’t that very visible sometimes.’ (Participant 5, female, 63 years)

‘E.g. the former Director of Finances did not want to put the lift in our building which was inaccessible for staff and students with disabilities … because he is one of those guys who squeezes a Rand until it starts to cry. … The other problem is budgeting. Say for instance, I submit a report for a building to be adjusted and maybe it’s not a building to be prioritized by our or according to our management, they prioritize building whose reports or audits were submitted five years back [*they will priorities this building after 5 years*] so now I come with a different building for them to see where to fit it, so it’s a whole prioritising thing.’ (Participant 2, female, 36 years)

‘There is no money lying around for things like that, if we see an urgent need then it’s a joint effort and we then try to get the money together, but there is not. For example, there is not each year, let’s say 5 million budget budgeted for the upgrade of infrastructure for disability access, that is not there so we do it as we see the need arise.’ (Participant 3, male, 36 years)

Costs are often seen as a reason to justify university management’s reluctance to make the built environment accessible for WUSs. Research disputes this contention (Policy Paper 2011:10). ‘Despite perceptions to the contrary, accessible design is inexpensive, with one study stating that making buildings accessible represents less than 1% of total construction costs’ (UNESCO 2009, cited in Policy Paper 2011:10). Participants in the present study pointed out that what makes accessible design become more costly is the fact that improper ramps and other amendments are built rather than doing things correctly in the first place in consultation with PWDs. Howell and Lazarus have criticised the association of rehabilitation or alterations and adaptations to the built environment of South African campuses with high costs as not grounded on valid evidence (Howell & Lazarus 2003:69).

Howell and Lazarus (2003) argue that

whilst there are obviously some cost implications for developing accessible facilities, both local and international experiences indicate that the creation of barrier-free environments are more about appropriate and informed planning and design than they are about costly additions and adaptations. (p. 69)

Therefore, in order to achieve this ‘it’s a matter of building in the right way from the start, so that everyone gets to contribute their different perspectives’ (National Property Board of Sweden [SFV] 2014:4). Expertise in UD is required as ‘without sensible decisions and intelligent procurement, nothing will change, which means the results are not up to scratch and mistakes are costly to fix afterwards’ (National Property Board of Sweden [SFV] 2014:4). As Katsui has argued, rather than a focus on the cost of accessibility and the ‘costly problems of individuals’, a reorientation is required to view accessibility as ‘an added value for the university rather than costly problems of individuals’ (Katsui 2009).

### Core facilities and services: libraries, toilets and transport

Proponents of inclusive education have highlighted the importance of prioritising the accessibility of facilities such as lecture theatres, libraries, toilets and modes of transport (Thomas 2012:58–59). Libraries are often prioritised when it comes to inclusive educational practice for wheelchair users. Apart from providing curb cuts, ramps and lifts which are central for WUSs to access buildings, it is also important that library interiors are accessible and easy to navigate, so that once inside a library, for instance, ‘aisles between the bookshelves’ are wide enough for a wheelchair user to browse books (Hall and Tinklin 1998:47).

In the 2014 *The South African Libraries 20 Years Review*, the South African Minister of Arts and Culture, Emmanuel Nkosinathi Mthethwa stressed that:

Libraries are places that must be open for everyone, catering for people with disabilities, rural citizens, the jobless and the incarcerated. Our society will only move forward when all our people experience an ever-increasing access to information in different kinds of formats. It is a challenge to keep up with the demand. (Department of Arts and Culture 2014:4)

The DUSMs interviewed for the present study pointed out that although funds might not be available to make every corner of a campus accessible immediately, universities were failing even to prioritise facilities that are of fundamental importance to being a student such as libraries. Participant 6 pointed out that often libraries are among the oldest of a campus’s buildings, which then means that the heritage argument comes into play even though this might mean that a wheelchair user’s access to the library is limited.

‘Now we are sitting with that problem for instance, our library, we cannot make changes in our library because it’s a heritage site, it was designed and won a world prize for its architecture, we are sitting with that thorny issue, we have to get permission and it does not have a lift even though we would like to make it more accessible, but it must not damage or impinge the fact that it won this prize for design which makes me very annoyed because, it won a prize for design yes, but that design did not accommodate persons with disabilities. There is a ramp, but nobody can use it, it’s too steep. There is a lift, but the lift only goes to the third floor. That’s one of the things we are sitting on, how we can provide more access to the library.’ (Participant 6, female, 63 years)

Equally fundamental to any human being’s ability to function in a public institution without having their dignity compromised is the need for accessible toilet facilities. Under South Africa’s National Building Regulations, provisions 4.12.1 to 4.12.4 provide for toilet facilities in public buildings and educational and workplace environments that are accessible to wheelchair users. Despite these provisions, most educational institutions in South Africa still do not provide toilets that are accessible to wheelchair users (Department of Education 2007; Losinsky et al. 2003).

Our participants criticised the poor design and construction of toilet facilities on campuses that make it hard for WUSs to have access to dignified use of toilet facilities.

‘And the most important thing is that if … a person can’t get to the toilet, then nothing is accessible in a hotel or in a dormitory or anywhere else and that is universal accessibility. To change or to build a wheelchair accessible toilet is a battle, like a battle from hell. Even if you go to your university you will see if there is a ramp most students will walk on the ramp. You go to a hotel there are only 4 rooms that are wheelchair accessible. It is so stupid to have that because if I have a team of 8 wheelchair tennis players or 12 now I have to put them in different hotels because only 4 can stay in one hotel. Nobody understands, oh no I can’t say nobody understands, but very few understand universal accessibility. You don’t have to build a toilet for a person with a disability, a wheelchair accessible toilet with a room that’s 3 by 3 metres. You can take that 3 by 3 metres and every toilet you build instead of making it 90 cm and 1 and half meter so that any wheelchair person and any person who can walk can use that toilet for disabled people. Why do you have one toilet for disabled people? Why can’t you have 10 toilets for everybody and anybody to use?’ (Participant 7, female, 52 years)

As noted by Agarwal ‘access is the key to inclusion’ (2012:56). Without access to appropriate facilities, WUSs face being inconvenienced by having to wait for a long time to use the one accessible toilet that might be on offer, which would not be the case if universities adopted the principle of UD that toilets are accessible to both wheelchair users and non-wheelchair users as a matter of principle.

‘Old buildings should be renovated in such a way to make them more accessible like ramps, enough parking, rest rooms facilities and really accessible rest rooms facilities, not stupid stuff because sometimes [*laughs*] I’ve seen in some places that they take a normal bathroom and they stick a disability thing on it and put a railing or something and it’s not accessible at all.’ (Participant 3, male, 36 years)

Transport is another core service that participants in the present study emphasised as central to fostering greater access for SWDs and for wheelchair users in particular. The accessibility to adapted transport for wheelchair users is provided for in Article 9 of the UNCRPD. South Africa’s National Student Financial Aid Scheme (NSFAS) caters for SWDs by providing non-means tested financial support to SWDs to study at one of the country’s 23 public HEIs (NSFAS 2012:3). Included are transport costs to and from campus (NSFAS 2013 cited in Ndlovu & Walton 2016:4). However, DUSMs pointed out that in many cases flexible and suitable transportation simply does not exist, which makes it difficult for WUSs to socialise with other students or to access campus at all after hours.

‘Our students are not on campus … a lot of our students are in private accommodation and that makes it very difficult for them to be part of the university’s social clubs because you know transport for them is quite a challenge. Therefore, if you want to have an event with all students from various campuses, you can’t. The transport is coming to pick them up at the specific time of the day and therefore they are not here in the evenings. If they can’t socialise, they can only socialise between breaks of classes, that is a problem. And we don’t have a shuttle service you know, we don’t have that. The other thing is that I agree, you know, we have a challenge to start a social group you know and get them involved. Oh no because of the transport because of the different locations.’ (Participant 8, female, 64 years)

As Participant 6 pointed out, WUSs cannot simply ‘hop onto’ public transport as they require transport that is suitable. Lack of appropriate transport can leave these students socially isolated and unable to access basic services that others take for granted such as shops and entertainment.

‘Look the challenge is not only for SWDs, but it’s the same for our SWDs as it is for our non-disabled students at the residences. They are far from any, ok it’s even worse for our SWDs because they can’t hop a public transport, taxis because we are far and isolated from shopping areas centers and CBD, so we are isolated. … So, for them to get to those areas, accessible transport is a problem. It just makes it difficult for our SWDs to get out there and go to a movie, or to go shopping. The other students can hop a taxi to go out. SWDs might find it difficult because we don’t have accessible transport for wheelchair users and that’s also something we are looking at.’ (Participant 6, female, 63 years)

Access to transport moreover is not simply a matter of being able to socialise but has wider implications for SWDs’ sense of autonomy and choice, which came through in Participant 8’s comments:

‘I think it happens most at residence life, so it’s very important that we communicate to the residences where students are involved to include them in activities because they do a lot of cultural and social events and those kind of things, but I do know that sometimes for our students it might feel like more effort. For example, if you take a blind student who goes to a residence function or maybe a formal or something that happens at a different venue. A blind student needs transport to get there. Ok, while it does not mean that a blind student has a friend who gives them a ride or and maybe, they do. They get to the function and maybe they are not enjoying themselves and they want to leave but they are dependent on somebody else to take them, so it might be beneficial to have a transport arrangement on campus for students who are blind. For example, after you have been to a function there is a driver to take them when they want to go home. So, they don’t really have a choice, they are forced to be stuck there until somebody takes them home.’ (Participant 8, female, 39 years)

In contrast, participants from campuses where flexible and appropriate transportation does exist commented on how significant this is as a feature of being able to foster inclusion in practice of SWDs.

‘Even ensuring that there is accessible transport for SWDs because we have four sites, SWDs may wish to visit other students on other sites just, just like any other student would do. So, we have transport system a little shuttle service that is able to provide them access with that. It’s for any academic related activities so if they have interviews or internship placements. Our students even if they want to go shopping to our nearest shopping centre or they have to go hospital for physiotherapy or chemo.’ (Participant 9, female, 37 years)

‘Whatever we plan on campus it’s open to them, we have the bus that transports SWDs, not very far, but around different campuses.’ (Participant 10, female, 49 years)

In addition to the provision of services like these themselves, which have the potential to significantly assist SWDs, as Participant 11 made clear, universities need to work to raise awareness, conscientise and sensitise the non-disabled campus community to questions of diversity and inclusion.

‘On a daily basis, the parking outside that is marked for disabled people, we usually clearly go and ask them personally to remove his car, I actually at one stage went and put the notice on the windscreen to tell him that he is not allowed to park there. That’s the obstacle, the barrier that we have to deal with everyday, parking space. People park there because they don’t realise, there is not enough awareness, I mean, it’s like the moment that you have experienced the thing [*being disabled*], you understand better.’ (Participant 11, female, 43 years)

### *Ad hoc* and ‘quick fix’ approaches and a lack of systematic consultation with persons with disabilities when designing built environments

One of the criticisms that DUSMs have of South African universities’ approaches to making campus environments accessible to PWDs is that the approach is often what they termed ‘ad hoc’ – responding on a case-by-case basis rather than taking a systematic and principled approach to inclusion. Moreover, existing approaches often fail to take into account the full range of disabilities with, for example, university personnel equating disability with ‘someone in a wheelchair’ at the cost of ‘awareness of the needs of people with other impairments’ (Chard & Couch 1998:608–621). When approaches to inclusion are ad hoc, they can prioritise what Singal (2005:6) refers to as that which is ‘easy to accommodate’ – focusing on physical access, distribution of aids and appliances, or infrastructure such as ramps, rather than more difficult to achieve change in processes like pedagogy, curriculum or attitudes. Likewise, Thomas (2005) argues that ‘money is thrown at very visible and easy areas. Shiny new ramps and rails are a suitable quick fix’ (p. 45). And as some of our participants pointed out, these interventions are often made with little proper consultation with PWDs themselves and the result is poorly designed facilities that do not really improve quality of access.

‘Our new residences that should take into account the new building regulations, and if there is a problem like we had at one new residence built two years back, they installed the ramp to get into the first floor where there is a lift and everything, but the ramp gradient was incorrect, it was too narrow, it was basically [*laugh*] a service ramp, it was good for [*laugh, laugh*] getting fridges ups and downs. We put in a complaint and they had to build a new one, we were like now you see what happened, in future see to it that if you do something do it right the first time otherwise you waste money.’ (Participant 3, male, 36 years)

Rather than ad hoc or quick fix approaches that address only the most visible aspects of inaccessibility, participants stressed the need for systematic consultation with users in order to ensure that processes of designing, building or rehabilitating old buildings serve PWDs appropriately.

‘There should have been a clause that accommodated the fact that the building should have been built from the first brick that was laid accessible for SWDs, for PWDs but it is not there still because there are areas that are not accessible. So, there is still things that needs to be done from top management so that when we contract a guy to come and do the building there must be one of the things must already say, you must get a consultant that will consult you and give them advice on how to make this building accessible. I have been there, my colleague Mitchell [*who is one of the DUSMs who uses a wheelchair*] … went there one day and she couldn’t even go in. That is not acceptable for me and I mean the builders could not have built an inaccessible place if there was a stipulation in the contract which came from policies which says all buildings must be accessible.’ (Participant 11, female, 43 years)

### Failure to create a sense of belonging for students with disabilities

In recent years, several scholars (see, for example, Allman 2013:3; Fredericks 2010) have highlighted the concept of belonging as an important need for students in universities if they are to be fully included. Following Manja Klemencic’s (2016) definition,

belonging refers to a student’s perceptions of intimate association with the university: to feel a central and important part of the university and a sense of ownership of their university, each of which fulfils their human need for inclusion, acceptance and efficacy.

As argued by Klemencic, students’ sense of belonging to their universities is central to their ‘academic success and, more generally, for a student’s subjective sense of well-being, intellectual achievement, motivation and even health’ (Klemencic 2016). South African scholars Engelbrecht and Green (2011) define ‘inclusive education as educational policies and practices that uphold the right of learners with disabilities to belong and to learn in mainstream education’ (p. 4). Moriña and colleagues have argued that learning environments should be as inclusive as possible not only for the purposes of fostering a sense of belonging for all learners, including SWDs, but also for fostering full participation in the learning process and offering equal opportunities and quality learning for all these students (Moriña, Cortés & Melero 2013, cited in Van Jaarsveldt & Ndeya-Ndereya 2015:2).

Our participants pointed to the need to achieve a sense of belonging for WUSs as one of the inclusion challenges that universities are not being vigorous enough in confronting.

‘For me, commitment is very important because you need leadership on this one because without leadership it becomes a challenge and so you need what I call home away from home… Everything needs to be accessible to SWDs every time, whenever, even if you are going to town, it must be easier going everywhere, you must or he or she must feel being able to do everything with the support from the university. The toilet, the study room, the library, everywhere. He must not feel like I’m disabled, he must feel like part of the university community.’ (Participant 1, male, 52 years)

As Participant 12 pointed out, often accessibility and questions of belonging are closely related. Where a person does not have ready access to social and educational settings, a sense of isolation and shying away from being involved in campus activities results and fostering belonging thus becomes difficult.

‘I don’t know why our SWDs don’t want to be involved in things on campus. I think it’s accessibility, accessibility to the buildings for those who are in a wheelchair.’ (Participant 12, female, 36 years)

For Participant 6 the location of the disability unit on the top floor makes it unlikely that WUSs will feel a sense of belonging and having their needs acknowledged and respected.

‘And first of all [*raising the voice*] we are in the second floor, I mean really. Second floor, lift broken, how do my students get to me, how can they come and write exams? If it’s first week of orientation, how can I get to them? I have to walk downstairs to meet with the students. Because of my disability I’m not allowed to climb stairs. One of my staff members also has a disability, she is not allowed to climb stairs too. Then what do we do? I can’t foresee us moving downstairs, but we have to.’ (Participant 6, female, 63 years)

According to Ginsberg and Wlodkowski (2009), an accessible university-built environment gives SWDs a sense of belonging in the form of ‘welcome[*ing*] a diversity of learners and cause them to feel safe, capable and accepted, thus enhancing their overall learning experience’ (cited in Van Jaarsveldt & Ndeya-Ndereya 2015:2). It is perhaps the opposite of being wholeheartedly welcomed into a place to be forced to enter or exit an educational or residential building from the back.

‘Inclusive education means that you reach all your students in your classroom. Outside the classroom, inclusive education is about creating a space where everybody feels welcome, so if you have the residence and you have the first floor accessible and there is not a lift to the second floor a student with a wheelchair won’t be able to, will never be able to visit his friends on the second floor, so his friends must come and visit him in his room all the time. And what also happens is that you have the first floor, the step and then have the dining hall. So now when a student in a wheelchair wants to move to the dining hall he has to go from the outside, so do you feel welcome there? If you have to use the back door, so to make them aware of removing that step to make the student move within a residence, to put in a lift or whatever, but it will cost money. And also, structural barriers that you have to remove and also attitudes.’ (Participant 8, female, 39 years)

‘Don’t make their entrance or exit from the building far from others. They must always make sure that they feel special. The same entrance that others are using, just next to it. Make sure that there is inclusion. Don’t put it there at the back or something, it’s another element of discrimination according to me. Make them feel at home. It’s like you are hiding them. Make sure that also the inclusion its where others will acknowledge them. It’s in a way that will give them dignity, some level of dignity to say that I’m also a human being. That’s why even here we don’t even think of them as differently abled.’ (Participant 13, male, 32 years)

For Claudine Sherill (2004), social inclusion has to do with, for example, students with and without disabilities interacting with one another reciprocally. Physical barriers compromise the prospect of these forms of reciprocal friendships flourishing, confining, for instance, WUSs to specific areas and spheres of movement and interaction.

## Concluding remarks

DUSMs know what ought to be done to make campuses accessible and welcoming to WUSs among other SWDs. However, their structural position in universities often means that they are powerless to influence policy implementation in practice. The goal of inclusive education is ‘supporting students with disabilities to be involved with their non-disabled peers to the maximum extent possible’ (Dalton et al. 2012:2). However, WUSs are still not being afforded full membership status at many South African universities and a lack of adequate access to such basic services as libraries, toilets and transportation makes it difficult for these students to participate fully in both the academic and social aspects of campus life and to reach their full potential. Often, resource constraints are invoked as the reason for why this is the case but, as Tania Burchardt (2004) has pointed out, ‘provisions necessary to meet the needs of people with impairments are demanded as a matter of right, rather than being handed out as charity to supposedly passive, grateful recipients’ (pp. 736–737).

The concept of ‘design for all’ when building new buildings or when rehabilitating old buildings ‘is an approach that means all products, environments and services are designed to function for as many people as possible’ (SFV 2014:4). The concept of design for all requires all key stakeholders such as architects, designers and accessibility officers and other experts to work in collaboration and consultation with PWDs in order to arrive at innovative solutions and achieve optimal results. South African universities are not yet at this point. Although some do collaborate with or consult with DUSMs in the building of new buildings or rehabilitating old buildings, DUSMs often occupy a marginal position in these consultations. Their relative powerlessness in the overall university hierarchy often means that their voice is occluded by other considerations such as heritage preservation or cost. As a result, commitments to UD and full inclusion often remain unrealised in practice.

There is a lack of consultation with, and taking seriously the views of, end users of core university facilities – such as libraries, toilets and transportation – without full access to which WUSs cannot flourish at university or feel welcomed in these environments. In order for HEIs to effectively identify factors that enable or hinder WUSs’ use of facilities, greater involvement and participation of the latter in decision making is critical: ‘Greater focus must be placed on listening to the voices of people with disabilities, to enable the development, implementation and evaluation of truly disabled friendly policies and programmes’ (Singal & Jeffery 2009:16; see also Imrie & Hall 2001:337). Those responsible for the planning, designing, adapting or upgrading of the built environment of campuses, especially architects, need to consult with SWDs for their input regarding their views, experiences and ideas, which are an essential component of creating genuine accessibility.

Rather than fully embracing UD and access as non-negotiable principles, we find South African universities framing access as one among a variety of competing interests in a context of scarce resources. This makes it difficult for DUSMs to have their voices heard or taken seriously in the implementation of practices that would result in universities’ built environments being truly accessible to WUSs. What we often see are quick fix solutions and ad hoc approaches rather than the systematic implementation of UD aimed at full access for all. Non-compliance with legal obligations attracts little in the way of sanction, and management routinely cite heritage preservation and cost implications as reasons for failing to take the necessary steps to make universities universally accessible and achieve the goal of full inclusion.

DUSMs can play a leading role in sensitising the non-disabled members of universities, both staff and students, in order to create greater awareness of, and appreciation for, the multiple ways in which WUSs continue to be excluded from full participation in university life. South African universities need to adopt a systemic approach to inclusion, which includes support staff, management and lecturers in the process of disability inclusion and fosters an understanding of inclusion as a fundamental right rather than as a luxury, which is dependent on affordability.

Inclusion, moreover, must extend not only to the academic aspects of student life but all areas of campus life including socialising. Although there is a need to prioritise fundamental services such as libraries, toilets and transportation, student life is also about sports, recreation, cafeterias, shopping and cinemas and WUSs are as entitled to full participation in these aspects of life as any other person. Respect for diversity requires that barriers to such full participation be removed, regardless of competing interests of the costs involved. Approaches to attaining this goal, moreover, need to be systematic, proactive rather than reactive and require a commitment on the part of the management and leadership of universities rather than relegating the sole responsibility for speaking up on behalf of SWDs to DUSMs who often occupy a position on the margins of power and influence in universities.

## Footnotes

1 Teletype device allows deaf people to type their messages instead of speaking, often abbreviated as TTY.

2 Convention on the Rights of Persons with Disabilities, 2006.

3 ‘The National Heritage Council of South Africa is a statutory body that is responsible for the preservation of the country’s heritage. Since its existence on 26 February 2004, it has managed to place heritage as a priority for nation building and national identity’ (National Heritage Council, 2016).

## References

[CIT0001] Agarwal A, 2012, ‘Steps towards inclusion: Access for all in higher education’, in *Enabling access for persons with disabilities to higher education and workplace: Role of ICT and assistive technologies*, Ford Foundation, viewed 5 October 2016, from http://www.nevertheless.in

[CIT0002] Allman D, 2013, ‘The sociology of social inclusion’, *SAGE Open* 1–16. https://doi.org/10.1177/2158244012471957

[CIT0003] Burchardt T, 2004, ‘Capabilities and disability: The capabilities framework and the social model of disability’, *Disability and Society* 19(7), 735–751. https://doi.org/10.1080/0968759042000284213

[CIT0004] Chard G. & Couch R, 1998, ‘Access to higher education for the disabled student: A building survey at the University of Liverpool’, *Disability and Society* 13(4), 603–623. https://doi.org/10.1080/09687599826632

[CIT0005] Dalton E.M., Mckenzie J.A. & Kahonde C, 2012, ‘The implementation of inclusive education in South Africa: Reflections arising from a workshop for teachers and therapists to introduce Universal Design for Learning’, *African Journal of Disability* 1(1), 1–7. https://doi.org/10.4102/ajod.v1i1.13 28729974 10.4102/ajod.v1i1.13 PMC5442567

[CIT0006] Department of Arts and Culture, 2014, *The South African libraries 20 years review*, Department of Arts and Culture, Pretoria.

[CIT0007] Department of Education, 2007, *National assessment report (public ordinary schools)*, National Education Infrastructure Management System, Contract EDO 305, Department of Education, Pretoria.

[CIT0008] Department of Higher Education and Training (DHET), 2013, *White paper on post-school education and training*, Government Printers, Pretoria, South Africa.

[CIT0009] DSD, DWCPD & UNICEF, 2012, *Children with disabilities in South Africa: A situation analysis: 2001–2011*, Department of Social Development/Department of Women, Children and People with Disabilities/UNICEF, Pretoria.

[CIT0010] Engelbrecht P. & Green L., 2001, *Promoting learner development: Preventing and working with barriers to learning*, Van Schaik Publishers, Pretoria. PMCid:

[CIT0011] Foundation of Tertiary Institutions of the Northern Metropolis (FOTIM), 2011, *Disability in higher education: Project report*, Ford Foundation University of the Witwatersrand, Pretoria.

[CIT0012] Fredericks B, 2010, ‘What health services within rural communities tell us about aboriginal people and aboriginal health’, *Rural Society* 20, 10–20. https://doi.org/10.5172/rsj.20.1.10

[CIT0013] Hall J. & Tinklin T, 1998, *Students first: The experiences of disabled students in higher education*, The Scottish Council for Research in Education Research Report No. 85, Scottish Council for Research in Education, Edinburgh.

[CIT0014] Gal E., Schreur N. & Engel-Yeger B, 2010, ‘Inclusion of children with disabilities: Teachers’ attitudes and requirements for environmental accommodations’, *International Journal of Special Education* 25(2), 89–99.

[CIT0015] Ginsberg M.B. & Wlodkowski R.J, 2009, *Diversity and motivation: Culturally responsive teaching in college*, Jossey-Bass, San Francisco, CA.

[CIT0016] Hendricks A, 2016, ‘Disabled UCT students demand access’, *Ground Up*, 27 January 2016, viewed 2 September 2016, from http://www.groundup.org.za/article/disabled-uct-students-demand-better-access/

[CIT0017] Howell C. & Lazarus S, 2003, ‘Access and participation for students with disabilities in South African higher education: Challenging accepted truths and recognising new possibilities’, *Perspectives in Education* 21(3), 59–74.

[CIT0018] Imrie R. & Hall P, 2001, ‘An exploration of disability and the development process’, *Urban Studies* 38(2), 333–350. https://doi.org/10.1080/00420980124545

[CIT0019] Katsui H, 2009, ‘Hisayo in “Accessibility for All at Higher Education” Seminar’, May 11, Disability Rights in Uganda-Research Blog, viewed 11 November 2016, from http://disability-uganda.blogspot.co.za/2009/05/hisayo-in-accessibility-for-all-at.html

[CIT0020] Klemencic M, 2016, ‘How to develop a sense of belonging’, *University World News*, 26 February, viewed 10 October 2016, from http://www.universityworldnews.com/article.php?story=20160223211126282

[CIT0021] Losinsky L.O., Levi T., Saffey K. & Jelsma J, 2003, ‘An investigation into the physical accessibility to wheelchair bound students of an Institution of Higher Education in South Africa’, *Disability and Rehabilitation* 25, 305–308. https://doi.org/10.1080/0963828021000043743 12745953 10.1080/0963828021000043743

[CIT0022] Lourens H, 2015, ‘The lived experiences of higher education for students with a visual impairment: A phenomenological study at two universities in the Western Cape, South Africa’, unpublished PhD thesis, Stellenbosch University, Cape Town.

[CIT0023] Matshedisho K.R, 2010, ‘Experiences of disabled students in South Africa: Extending the thinking behind disability support’, *South African Journal of Higher Education* 24(5), 730–744.

[CIT0024] Moriña A., Cortés M.D. & Melero N, 2013, ‘Inclusive curricula in Spanish higher education? Students with disabilities speak out’, *Disability and Society* 10, 1–14. https://doi.org/10.1080/09687599.2013.769862

[CIT0025] Mitchell D, 2010, *Education that fits: Review of international trends in the education of students with special educational needs*, final report, University of Canterbury. Ministry of Education, Wellington, New Zealand.

[CIT0026] National Heritage Council, 2016 Our Mandate. Department of Arts and Culture. Accessed 10 May 2017, from http://www.nhc.org.za/about-us/mandate/

[CIT0027] National Student Financial Aid Scheme (NSFAS), 2012, *Annual report 2012*, NSFAS, Cape Town.

[CIT0028] National Student Financial Aid Scheme (NSFAS), 2013, *Guidelines for students with disabilities for the Department of Higher Education and Training Bursary Programme*, NSFAS, viewed 6 November 2016, from http://www.org.za/NSFAS/STUDENTS/BURSARIES

[CIT0029] National Property Board of Sweden (SFV), 2014, *Dignified entrance*, National Property Board, National Property Board of Sweden (SFV), Stockholm, Sweden.

[CIT0030] Ndlovu S. & Walton E, 2016, ‘Preparation of students with disabilities to graduate into professions in the South African context of higher learning: Obstacles and opportunities’, *African Journal of Disability* 5(1), 1–8. https://doi.org/10.4102/ajod.v5i1.150 10.4102/ajod.v5i1.150 PMC5433445 28730040

[CIT0031] Parliamentary Monitoring Group, 2015, *South African Heritage Resources Agency & National Heritage Council: Legislative mandates and roles in identification, conservation and management of cultural resources*, Parliamentary Monitoring Group, Cape Town.

[CIT0032] Policy Paper, 2011, *Making inclusive education a reality*, viewed 13 August 2016, from www.sightsavers.org

[CIT0033] Sherill C, 2004, *Adapted physical activity: Recreation and sport: Cross disciplinary and lifespan*, McGraw-Hill, Dubuque, IA.

[CIT0034] Singal N, 2005, ‘Responding to difference: Policies to support “inclusive education” in India’, paper presented at the Inclusive and Supportive Education Congress 1–4 August, 2005, University of Strathclyde, Glasgow.

[CIT0035] Singal N. & Jeffery R, 2009, ‘Transitions to adulthood for young people with disabilities in India: Current status and emerging prospects’, *Asia Pacific Disability Rehabilitation Journal* 20(1), 15–40.

[CIT0036] South African Human Rights Commission, 2012, *Submission to the Portfolio Committee and Select Committee on Women, Children and People with Disabilities: Implementation of the Convention on the Rights of Persons with Disabilities (CRPD)*, South African Human Rights Commission, Johannesburg.

[CIT0037] Thomas A.S, 2012, ‘Disability through a human rights paradigm’, in *Enabling access for persons with disabilities to higher education and workplace: Role of ICT and assistive technologies*, Ford Foundation, viewed 4 October 2016, from http://www.nevertheless.in

[CIT0038] Thomas P, 2005, *Mainstreaming disability in development: India country report*, Disability Knowledge and Research, London, viewed 4 October 2016, from http://disabilitykar.net/research/polindia.html

[CIT0039] UNESCO, 2009, *Policy guidelines on inclusion in education*, viewed 13 August 2016, from http://unesdoc.unesco.org/images/0017/001778/177849e.pdf

[CIT0040] Van Jaarsveldt D.E. & Ndeya-Ndereya C.N, 2015, ‘It’s not my problem’: Exploring lecturers’ distancing behaviour towards students with disabilities’, *Disability and Society* 30(2), 1–16. https://doi.org/10.1080/09687599.2014.994701

[CIT0041] Wolanin T.R. & Steele P.E, 2004, *Higher education opportunities for students with disabilities*, The Institute for Higher Education Policy, Washington, DC, viewed 4 November 2016, from http://www.ihep.org

[CIT0042] World Health Organization (WHO) & The World Bank, 2011, *World report on disability*, viewed 8 August 2016, from http://whqlibdoc.who.int/publications/2011/9789240685215_eng.pdf

